# Axial slices compared to short-axis slices for measurement of right and left ventricle volumes of patients with corrected Tetralogy of Fallot and Ebstein's anomaly by cardiac magnetic resonance

**DOI:** 10.1186/1532-429X-11-S1-P210

**Published:** 2009-01-28

**Authors:** Sohrab Fratz, Annika Schuhbaeck, Christina Buchner, Stefan Martinoff, John Hess, Heiko Stern

**Affiliations:** Deutsches Herzzentrum München, Munich, Germany

**Keywords:** Congenital Heart Disease, Cardiac Magnetic Resonance, Axial Slice, Left Ventricle Volume, Ventricle Volume

## Objective

To determine if axial slices or short-axis slices should be the method of choice for routine clinical measurement of right (RV) and left (LV) ventricle volumes of patients with congenital heart disease (CHD) by cardiac magnetic resonance (CMR).

## Background

RV and LV volumes and function need to be measured reliably by CMR in the long-term follow-up of patients with CHD. However, no standard protocol to measure RV volumes by CMR exists. A standard protocol, LV short-axis slices, exists only for the LV. However, the basal boundary of the RV is difficult to trace using LV short-axis slices. Axial slices through the patient's chest have been suggested as an alternative. Axial slices are potentially beneficial compared to short-axis slices. Firstly, axial slices are very easy to plan. This is especially true in complex anatomy, as is often the case in CHD. Secondly, by using axial slices valuable morphologic information about the large vessels and atria is obtained without need of further scans. It is known that in normal individuals, RV volume measurements made from axial slices are feasible and have a superior reproducibility to conventional short-axis slices. However, using axial slices measuring RV volumes in patients with RV morphology and pathology has not been tested. Additionally, the reproducibility of measuring LV volumes from axial slices has never been studied, neither in normal individuals nor in patients with CHD. Therefore, taken together, to date the best slice orientation for the routine clinical measurement of RV and LV volumes in patients with CHD remains unknown.

Therefore, the aim of this study was to determine if short-axis or axial slices should be the method of choice for the routine clinical measurement of RV and LV volumes in patients with CHD.

## Methods

We studied two homogenous patient groups with RV pathology (corrected Tetralogy of Fallot, n = 46 and Ebstein's anomaly, n = 15) in which both axial slices and short-axis slices can easily be obtained by CMR. By using the f-test the inter- and intra-observer variances of both methods were compared for four volumes (enddiastolic and endsystolic RV and LV volumes) in both groups.

## Results

Eight variances were significantly smaller in the axial data sets compared to the short-axis data sets. The remaining eight variances were not significantly different from another.

## Conclusion

Routine clinical measurements of RV and LV volumes by CMR in patients with corrected Tetralogy of Fallot and Ebstein's anomaly should be aquired from axial slices and not short-axis slices. We furthermore suggest that axial slices should also be the method of choice for the routine clinical measurement of RV and LV volumes in all other forms of CHD.

